# Dr. Pindell

**Published:** 1833

**Authors:** 


Dr. Richard Pindell, of *Lexington, Ky.* This aged veteran in the profession, expired on the 20th of March, in his 79th year. He was a native of Maryland, but had practised his profession at Lexington, for the last twenty years. In sound practical skill, devotion to business, benevolence to the poor, and urbane and gentlemanly deportment, Dr. Pindell was surpassed by few members of the profession in the West; and will be long remembered with gratitude, by an extensive circle of patients and friends, both in Maryland and Kentucky. The editor of the Transylvania Journal has promised his readers a biographical sketch of Dr. P. to which we shall refer in a future number.

